# Sustainable Solutions for Plastic Waste Mitigation in Sub-Saharan Africa: Challenges and Future Perspectives Review

**DOI:** 10.3390/polym17111521

**Published:** 2025-05-29

**Authors:** Comfort Yeboaa, Emmanuel Kweinor Tetteh, Martha Noro Chollom, Sudesh Rathilal

**Affiliations:** Green Engineering Research Group, Department of Chemical Engineering, Faculty of Engineering and the Built Environment, Durban University of Technology, Durban 4001, South Africarathilals@dut.ac.za (S.R.)

**Keywords:** biodegradation, bioremediation, fungi, microbes, plastics, Sub-Saharan Africa

## Abstract

The anthropogenic deployment of plastic waste, especially petroleum-based plastics with toxic hydrocarbons, presents a significant environmental and health threat in sub-Saharan Africa (SSA). Herein, the high demand and rapid plastic production, coupled with improper disposal and inadequate waste management, have led to widespread contamination of air, water, and soil. Conventionally, plastic waste management, such as incineration and recycling, provides limited long-term solutions to this growing crisis. This necessitates urgent, sustainable, and eco-friendly remediation techniques to mitigate its far-reaching environmental implications. This comprehensive review focused on sustainable and eco-friendly techniques by exploring strengths, weaknesses, opportunities, and threats (SWOT) analysis of plastic waste management. Bioremediation techniques were found as potential solutions for addressing plastic waste in SSA. This paper examines advancements in physiochemical methods, the challenges in managing various plastic types, and the role of enzymatic and microbial consortia in enhancing biodegradation. It also explores the potential of genomic technologies and engineered microbial systems to convert plastic waste into valuable products, including bioenergy via bio-upcycling. These bioremediation strategies align with the United Nations Sustainable Development Goals (UN SDGs), offering a promising path to reduce the environmental and health impacts of plastic pollution in the region. This paper also considers future directions of integrating AI-powered recycling systems to facilitate the development of a circular economy in SSA. Additionally, this paper provides progress and future perspectives on bioremediation as a sustainable solution for plastic waste management in SSA.

## 1. Introduction

The global production of plastic over the last 20 years was estimated to be 335 million tons in 2021, which is anticipated to upsurge to about 600 million tons by 2030 if action is not taken against it [1,2]. In essence, plastics are generally used for packaging for food and beverages, sports, medical equipment, electrical equipment, and other household wares. In sub-Saharan Africa (SSA), the annual plastic importation and production is approximated to be around 117.6 million tons, whereby 80% ending up as solid waste poses a greater threat to the environment than its economic value [2,3]. Herein, the uprising of plastic waste in the ocean and environment has adverse effects on both human health and marine life [1,2]. Additional effects on animals include entanglement and starvation (e.g., altered behavior or reduced fitness) [4,5]. Also, long-term ingestion of plastics (e.g., microplastics) by humans may result in genetic abnormalities, obesity, infertility, and cancer [1,6].

Despite the growing awareness of the dangers of plastic waste in the environment and ocean, 8 to 10 million metric tons of plastic waste are produced annually [2,3]. According to Hira et al. [7], it is anticipated that by 2050, there will be more plastic in the sea than there will be fish. This is because plastics do not degrade completely, and degradation could take about 500–1000 years [8]. Although knowledge of advanced analytical techniques in the detection of emerging pollutants and plastics in the environment is growing, their complete degradation is still not comprehended [9,10]. This has become a great concern about the planet’s use of plastics as there are no substantial policies or sustainable frameworks for mitigating plastic waste [11]. Therefore, comprehensive knowledge and critical action are required to mitigate plastic waste challenges for a sustainable environment and save the ecosystem from the detrimental effects of plastic waste.

Plastics are generally categorized into thermoplastics and thermosets; thermoplastics account for over 80% of the plastics used globally. However, the chemical properties and usage profiles of both thermoplastics and thermosetting polymers derived from petroleum-based feedstocks present considerable challenges in recycling and waste management [3,8,9], such as improper disposal and inefficient disposal planning [12,13]. With regard to mitigating the uprising of plastics in the environment, the most conventional practice has been burning/incinerating. However, dangerous chemicals such as carbon monoxide, nitrogen oxides, and chemicals like dioxins are emitted from burning the plastic, which are detrimental to the health of residents [14,15]. The recycling of plastics has also been identified as a method of mitigation. However, less than 10% of plastics are recycled [16]. Therefore, it is essential to develop eco-friendly waste management systems to save the ecosystem from the detrimental impacts of plastic waste.

Recent research emphasizing the potential for microbial breakdown of plastic waste as a long-term solution has demonstrated to be viable [16,17,18]. This, as well as bioremediation, is becoming desirable since it can rapidly deteriorate plastic waste into metabolic products (CO_2_ and H_2_O) [19,20,21]. However, the scarcity of data and information on the fate and related toxicological impacts of plastic waste in SSA affects most of the country’s capacity to devise policies for the mitigation of pollution [22,23,24]. The SSA, with an economy of 48 countries, is dominated by the unregulated market usage of plastics for packaging smaller retail products at cheap prices and volumes. Additional usage includes the paucity of water infrastructure, and transporting fresh drinking water by plastic has become very crucial. However, SSA represents 13% of the global population (>1.17 billion) and is projected to increase by 22% (2.2 billion) by 2050 [25,26]. Notwithstanding the global economic and industrial transformation, the urban population is anticipated to increase by 50% by 2035 [25]. This gives an advantage to SSA for economic growth and development with an increase in population to boost the labor force for the high demand for products and services. Despite the region’s sluggish economic growth and unemployment, it is also confronted with severe plastic waste pollution threats to the ecosystem and its biodiversity. Reports show that about 180 metric tonnes of waste are generated in the region, with 0.5 kg/day of waste produced per person [16,27,28]. Also, most countries in SSA are undeveloped countries, where about 50% of the population lives below the poverty line of “USD 1.25 per day” [29,30]. Moreso, waste production is competing with population growth, and the lack of standardized facilities for plastic waste collection poses potential risks to the environment and human health. Nevertheless, the region’s lack of adequate waste management infrastructure and strong plastic market demand have caused plastic waste to become more prevalent. Hence, mapping out strategies to mitigate waste research focus areas in SSA comes in handy.

In this comprehensive review, current trends of published articles on plastic waste as a threat to human health and the environment, notably in the SSA region, are discussed. Furthermore, challenges associated with conventional plastic waste mitigation technologies and biodegradation in terms of identifying the most influential abiotic and biotic mechanisms are highlighted [28]. The microbial variety and bioremediation mechanisms that may be intrinsically adapted for the degradation of high-molecular-weight polymers and promote a biological up-cycling solution of plastic waste were explored. Additionally, the setbacks of the UN sustainability development goal to the environmental, health, and economic impact of plastic usage were presented. Additionally, the prospects of AI-powered plastic recycling as a sustainable solution were highlighted.

## 2. State of Plastic Waste Management Research

**Figure 1:**
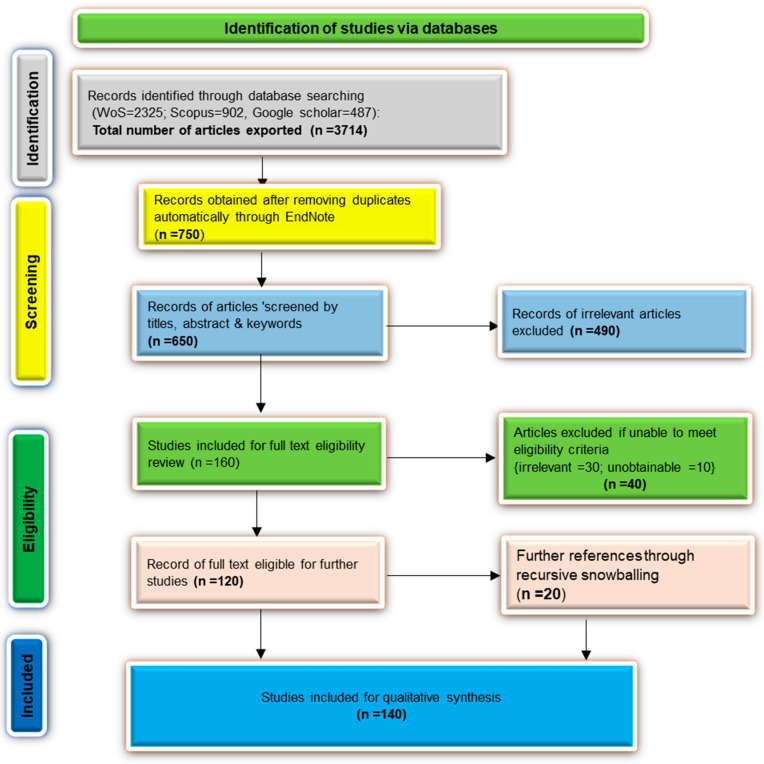
Schematic database collection and PRISMA framework for the study.

A desktop study provided a strong trend and progress on plastic waste management with about 3714 published articles from the Web of Science (WoS), Scopus (SC), and Google Scholar (GS) databases within the period 2010–2023. A scoping review technique, such as Preferred Reporting Items for Systematic Reviews and Meta-analyses (PRISMA), was then employed to evaluate the literature’s depth and breadth (Figure 1). Under this scope, a four-step approach addressed some of the study concerns within plastic waste engineering and management. These steps included (i) identifying the research papers, (ii) identifying pertinent studies, (iii) selecting and extracting information, and (iv) data analysis and reporting the findings. The research gap on the mitigation of plastic waste within the SSA region was identified via a methodical search of published articles for empirical studies that assisted in identifying the limitations and novel approaches to mitigating plastic waste pollution. A recommendable future trajectory that is effective and environmentally friendly for applied research in the SSA region was then discussed. Using the Web of Science (WoS), Scopus (SC), and Google Scholar (GS) databases, the search between 2010 and 2023 provided authoritative and high-quality research findings. Keywords and phrases with Booleans (AND, OR) in the field tags search, such as topic (TS) and all fields (ALL), were used (Table S1). This study was limited to English-language and published articles (35% research and 65% review articles) with open access. The papers were then exported in the RIS format to the EndNote program for proper reference management. At this step, duplicated journals were automatically screened and removed from the database using the EndNote tool and the article titles. Studies that did not align with the objectives and scope of this review were excluded through an iterative screening process. The eligibility of articles was then determined by gradual screening and modification via the framework depicted in Figure 1. The total number of articles was reduced to 120 and evaluated for their content.

### 2.1. Annual Publication Trend on Plastic Waste

**Figure 2:**
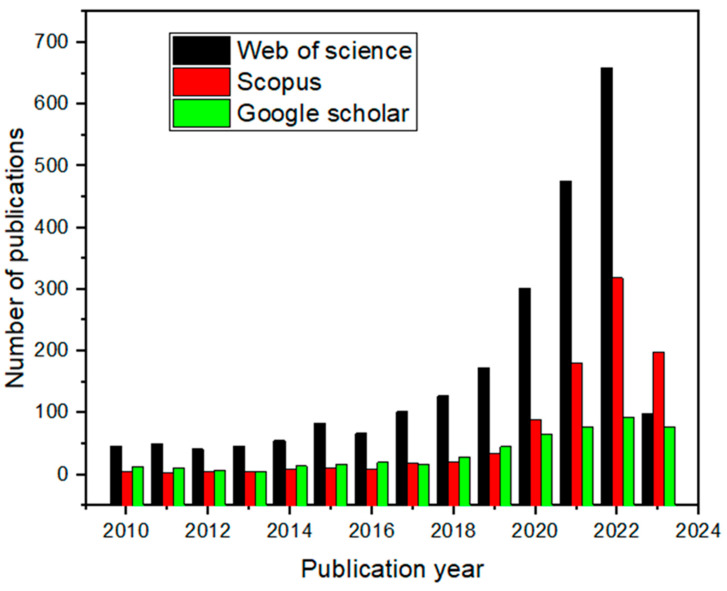
Publication trend of plastic waste from 2010 to 2022 obtained from Web of Science (WoS), Scopus (SC), and Google Scholar (GS) database.

The selected full-text studies evaluated for qualitative synthesis spanned from 2010 to 2023 and were not limited to 2024 and 2025, proving that the concept of plastic pollution research was prevalent. The information obtained from the papers was then elucidated to highlight the SSA findings. This was carried out to ascertain information on the subject matter and its relationship with SSA findings. Figure 2 shows the publication trend on plastic waste management concerning published papers from databases such as Web of Science (WoS), Scopus, and Google Scholar (GS). The publication trend revealed that emerging scientific research contributions were recorded in 2014; after that, more scientific contributions were made towards the subject development. Subsequently, there is a giant leap in the 2021 poll of publications until 2022, then a drop in 2023. The WoS database (Figure 2) revealed that emerging scientific research contributions were recorded in 2014; after that, more scientific contributions were made towards the subject development. Subsequently, there is a giant leap in the 2021 poll of publications until 2022. This could be linked to the continual emphasis on the global sustainability of the ocean and the need to establish recyclable plastics [17]. The evaluation also highlighted potential plastic waste solutions, the advantages of plastic waste management, public participation, and perspectives regarding plastic waste management. It was found that the discussion on persistent plastic waste propagation and its associated challenges has gained global research attention. Most of the papers reviewed in this study were from diverse regions across the world, whereas the number of papers published in Africa was very limited. The evaluation also highlighted potential plastic waste solutions, the advantages of plastic waste management, public participation, and perspectives regarding plastic waste management. It was found that the discussion on persistent plastic waste propagation and its associated challenges has gained global research attention.

### 2.2. Authorship Regional/Country Occurrence

**Figure 3:**
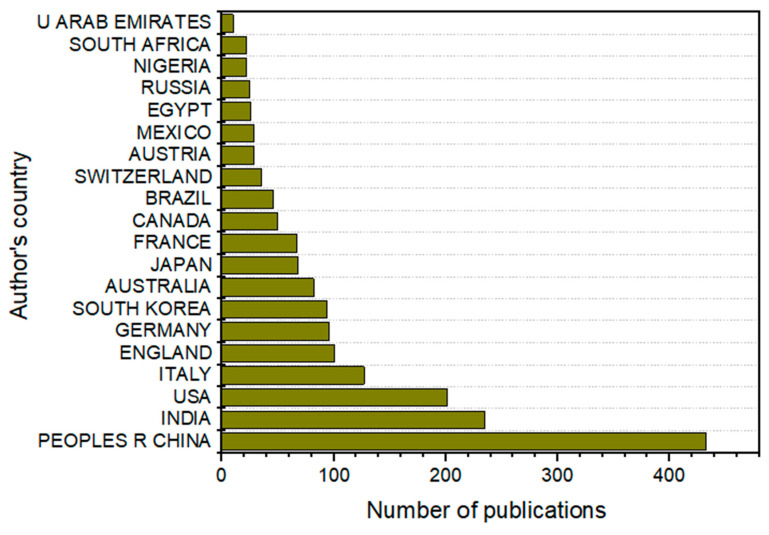
Author’s publication on plastic waste management affiliated countries.

The EndNote reference management software revealed the published papers affiliated with individual authors (Figure 3). Most of the published papers reviewed in this study were from diverse regions worldwide, whereas the number of papers published from the SSA region was very limited. The People’s Republic of China was the most affiliated country and had the most collaborative publications (25%) with other countries. With its industrious economy, China has a national roadmap that bridges the gap between academia and industries for socioeconomic growth and development. It has also paid attention to international partnerships, grants, and opportunities for postgraduate research collaboration. The high research collaborations at the global level were also demonstrated by contributions from China > India > the USA > Italy > England > Germany > South Korea. This was followed by Australia > Japan > France > Canada > Brazil > Switzerland > Austria > Mexico. The small sample size of published papers (70) on plastic waste management in Africa compared to the rest of the globe may be attributable to the decision to restrict the study to SSA. There were limited contributions from SSA countries, like Egypt (26), Nigeria (22), and South Africa (22), which were among the first 20 countries. This requires urgent research attention, as SSA is recognized as the largest market hub for plastic production and waste generation. The challenges associated with plastic waste in SSA, the advantages of managing plastic waste, the health-related concerns, and the mitigating techniques were then explored.

### 2.3. Document Analysis and Plastic Research Interest

The analysis of the document revealed research development for plastic waste management with sustainable solutions. Most of the research field included but was not limited to environmental sciences ecology (992), engineering (679), polymer and material science (274), science and technology (272), biotechnology (194), energy fuels (102), microbiology (84), biochemistry(60), water resources (45), agriculture (40), marine freshwater biology (39), public environmental occupational health (32), food science technology (20), toxicology (18), construction building technology (13), and other disciplines (663). This assisted in developing a research interest in plastic waste management and its impact on the environment and the setback to the UN SDGs. The multidisciplinary research areas, such as engineering and science and technology, provided insight into plastics’ technological and production challenges. Meanwhile, material science, chemistry, and biochemistry have given insight into plastic properties and the potential to explore biodegradation techniques. Likewise, construction, food, and medicine were related to the application of plastics. It was deduced that most of the journal’s research interest in material science, medicine, water, and environmental engineering focused on the toxicological impact of plastic pollution on human health and the ecosystem.

#### 2.3.1. Keyword Analysis on Plastic Waste Management

**Figure 4:**
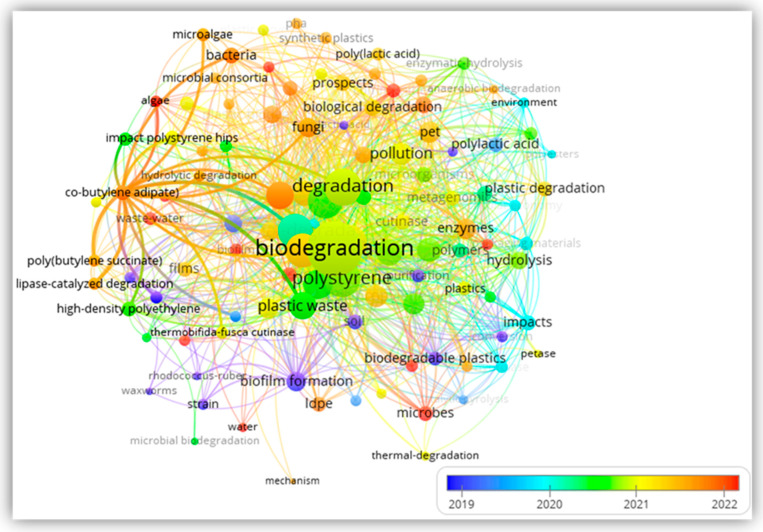
The overlay visualization of publication keywords strength.

The author’s keywords of the refined published papers (120) strongly linked to plastic waste management were analyzed by setting the considerable occurrence to a minimum of 20 to eliminate duplications. Among the 150 strong keywords, biodegradation was the most common, with 31 occurrences and a linked strength of 120. Microplastics (47 linked strength), microbial degradation (40 linked strength), enzymatic degradation (34 linked strength), and bioremediation (27 linked strength) were among the keywords that assisted in developing the research interest. Figure 4 shows the overlay visualization of the cluster-linked keyword’s strength with the publication’s year color codes. It was found that most of the dominant keywords were associated with publications from 2019 to 2022.

#### 2.3.2. Plastic Waste Management Research Themes

**Figure 5:**
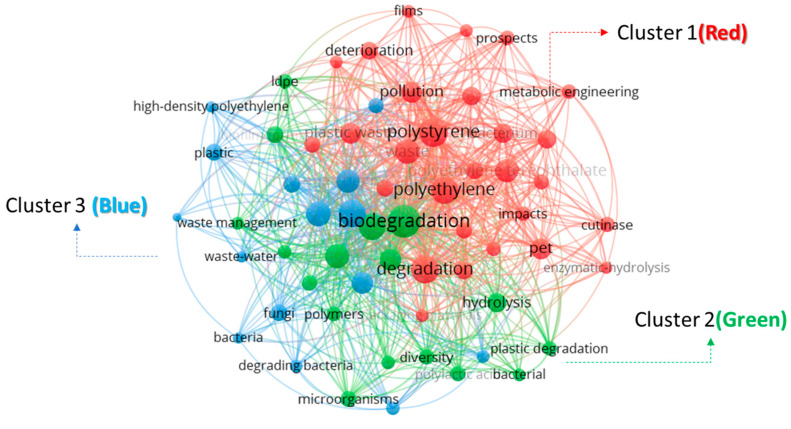
Cluster network of keywords associated with plastic waste management.

Research themes for plastic waste management were developed by analyzing the keywords’ occurrence as clusters. Figure 5 shows three main clusters from the keywords occurring in networks with distinct color codes. Cluster 1 (Red) had 70 keyword terms with strong relationships with mineralization and the metabolic engineering of plastics. It eludes the prospects of bioremediation of plastic waste and curbing its environmental impact. Cluster 2 (Green), with a network of 45 keywords, revealed the degradation mechanisms of plastic waste. This included biodegradation, microbial degradation, photodegradation, thermolysis, bioaccumulation, and biosorption. The role of bacteria in the enzymatic degradation of plastic waste was also revealed. Cluster 3 (Blue), with 35 keywords, revealed types of plastic, microorganisms, bacteria, polymers, and eco-friendly techniques for managing plastic in wastewater sources. Table 1 summarizes some publications associated with research themes and cluster network keywords.

## 3. Global Plastic Waste Management Mitigation

**Figure 6:**
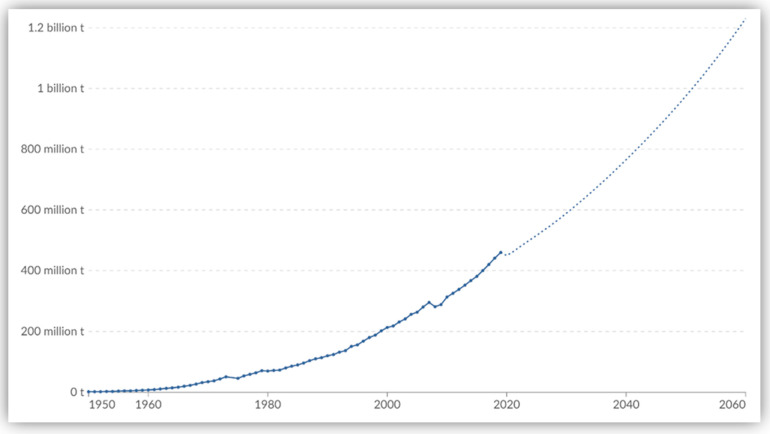
Global trend of estimated plastic waste production projected from 1950 to 2060 (data retrieved from https://www.statista.com/statistics/282732/global-production-of-plastics-since-1950/, accessed on 24 May 2025).

Historically, plastic manufacturing has expanded since 1950 (1.5 million metric tons), as illustrated by Figure 6, with the current robust trend. In 2021, the global output of plastics was estimated at 390.7 million metric tons, with a rise of 4% annually [25,27]. According to OECD [25,26], the global market index is expected to reach a value greater than USD 810 billion by 2030, with a compound annual growth rate of 3.7%. More than 50% of global virgin plastics manufactured for single purposes are discarded as waste after being utilized once [26,28]. However, by identifying and synthesizing plastics, the ability to endure high temperatures can be categorized as thermoplastics and thermosets [51]. In most applications, thermoplastics are linear or branched polymers that can be revamped within a wide range of temperatures [26,28] while thermosets are polymers with cross-links that are generally tough and unbreakable [25]. In addition, synthetic plastics, including polyethylene terephthalate (PET), high-density polyethylene (HDPE), polyvinyl chloride (PVC), low-density polyethylene (LDPE), polypropylene (PP), and polystyrene (PS), are among the seven divisions of the two main categories of plastics [5,26,51]. Notwithstanding the production types of plastics, the use and end-of-life management of polymer resins and synthetic fibers and additives present global challenges to be addressed.

**Figure 7:**
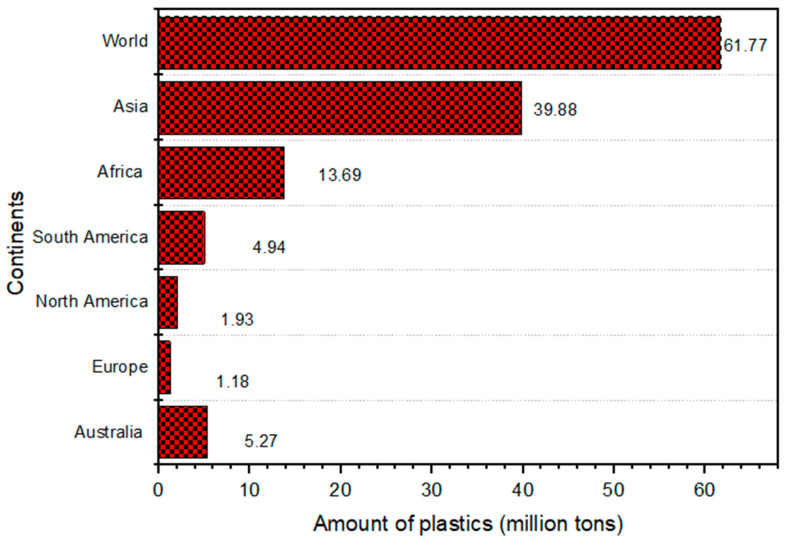
Estimated 2019 global plastic waste emitted into the oceans, adapted from [26].

Furthermore, municipal governments in many SSA countries lack regulatory support for plastic waste schemes [22,52]. The mismanagement of plastic waste coupled with poor economic growth in the SSA region creates poverty, worsened by a rising population [53]. This affects municipal waste streams since citizens and local governments simply cannot afford world-class services, making sustaining services problematic [54]. Lack of understanding across layers or arms of government, where waste management is not devoted to local governments, makes it difficult for citizens to receive effective waste management services, resulting in improper plastic waste management [22]. Figure 7 shows that the mismanaged plastic waste on the SSA was estimated in 2019 by either littering or inadequate disposal. According to Eriksen et al. [55], there are roughly 269,000 tonnes of plastic in surface waterways worldwide. This was estimated based on the capital plastic waste generated before waste management via either recycling or incineration without the pollution risk to waterways.

### 3.1. Sub-Saharan Africa’s Challenges in Managing Plastic Waste

Plastic enterprises in SSA are facing impediments, including inadequate plastic production by local industries that promote the importation of more raw materials than finished products. Due to the high exchange rate, continual importation has strangled many developing economies [53,56]. Also, the lack of raw material diversification has hampered the sustainability of most local industries. In several SSA countries, the lack of suitable facilities for bulk production and diverse applications has led to the monopoly use of most plastics produced [5,57]. Single-use plastic eventually becomes waste plastic, and the lack of knowledge of how to manage plastic waste makes it unsustainable. Changes in consumption patterns, rapid population expansion, increased urbanization, economic growth, ineffective collection, transportation, handling, and disposal are additional challenges [7,15,56]. Littering from unauthorized disposal of plastic waste or disposal facilities is frequent in SSA and harmful to human health and the environment. Due to its non-biodegradable nature, plastic waste pollutes surface and underground water, harming wildlife and the ecosystem [8,57]. Despite the inadequate management of municipal solid waste and plastic waste, the constraints that SSA faces vary in their degree of development across the region [7,22]. Some of these developmental challenges include a lack of facilities and experience with waste management issues, an unfavorable business and governance framework, and gender imbalances [22,57].

As shown in Figure 7, the SSA region generates about 14 million tons of plastic waste, of which 80% is mismanaged in comparison with the global tonnage of plastic waste produced in 2019 [26]. However, as projected in Figure 6, it is anticipated that the tonnage of plastic waste in SSA will also triple by 2060 [47]. Table 2 underscores the need for improved plastic waste management infrastructure and policies across SSA to handle the growing plastic waste trade effectively. South Africa stands out as the largest importer (USD 2.63 billion) and a significant exporter (USD 83.6 billion) of plastic products within SSA, indicating a more developed plastic industry and trade network [58]. However, the poor management of plastic waste from SSA shipping and marine activities, such as aquaculture and fishing, also ends up in the oceans. Whereas countries like Ghana, Ethiopia, Nigeria, Algeria, and Mozambique have varying degrees of import reliance, Kenya has implemented stringent policies to curb plastic usage [22,58].

#### 3.1.1. Industrial and Domestic Demand

**Figure 8:**
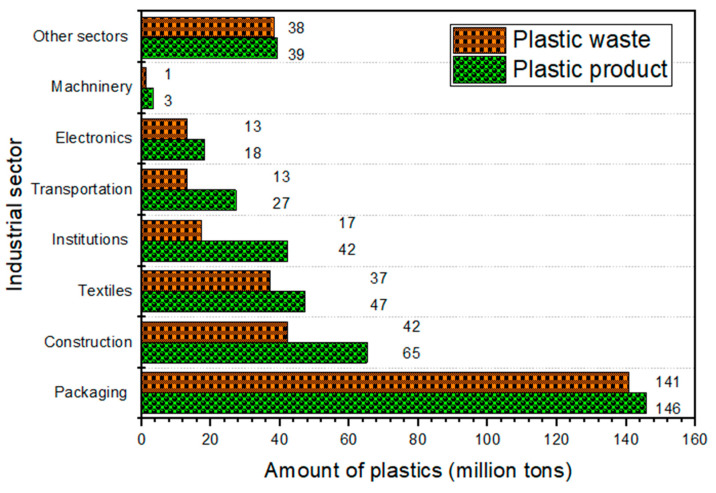
Estimated plastic demand products and waste produced in SSA, adapted from [22,26].

In SSA regions, the production of plastic and its waste management has both economic and environmental benefits [29]. This is due to the correlation between a rise in plastic production and waste generation in a market economy. Figure 8 indicates that human activities affect the demand for plastic products and the waste produced, which has detrimental health and environmental consequences [22].

As shown in Figure 8, plastics produced (387 million tons) are used for packaging (146 million tons), construction (55 million tons), textiles (47 million tons), institutions (42 million tons), transportation (27 million tons), electronics (18 million tons), machinery (3 million tons), and other sectors (39 million tons) [25,26]. Despite this, the polymer type and product lifetime of plastics can also influence the production of plastic waste. For example, packaging plastics have a short “in-use” lifetime, whereas building and construction polymers might have an average lifetime of over 35 years [25]. This makes packaging plastics the largest source of plastic waste (141 million tons), accounting for about 50% of the world’s total waste. Herein, waste management and policymakers are concerned about providing facilities to manage plastic waste in SSA. To mitigate these issues, the examined papers signpost plastic waste management via microbial and bioremediation should be given research attention, as limited knowledge is available, especially in SSA.

#### 3.1.2. Environmental and Health Risks of Plastic Waste in SSA

Plastic products are generally produced using additives, such as plasticizers, flame retardants, stabilizers, and antibacterial agents [14,28]. This causes plastic waste to contribute to the production of persistent organic pollutants. Other pollutants, such as polychlorinated biphenyls (PCBs), organic pollutants, and polyaromatic hydrocarbons (PAHs), are also associated with them [54,59,60]. Plastics have hydrophobic qualities due to the plasticizer and catalyst residues left behind from their manufacturing process, which can seep into the soil [55,61]. Additionally, plastic waste can contaminate the groundwater through several other pathways, such as sludge, fertilizers, wastewater irrigation, landfilling, biosolids, or other sources [25,26,62]. The persistence of plastic pollution in bodies of water is a major concern since it poses significant threats to the health and appearance of organisms [55,63]. Plastic waste, which contains carcinogens, can influence the food chain through plankton, fish, and ultimately, human health [64]. The chemical composition of plastics may have multiple effects on human health, such as the desorption of hydrophobic organic contaminants and the leaching of unbound chemicals and/or residual monomers [2,64,65]. The abundance of microplastics in *helicopter leucisculus* digestive tissue was determined to be between 2.3 and 15.8 items per gram of digestive tissue [6]. Mahmud et al. [66] also emphasized the histopathological damage induced by plastic contamination in fish tissues. This means that the inhalation and digestion of plastic particles can affect both aquatic and human life directly or indirectly. For instance, the persistence of microplastics can generate several biological reactions, such as inflammation, genotoxicity, apoptosis, oxidative stress, necrosis, tissue brain damage, fibrosis, and oncogenesis [1,6]. Due to the mishandling of plastic waste and the global effects of plastic waste, novel ways for treating and disposing of plastic waste are urgently needed. Additionally, consuming fish containing these hazardous substances raises the risk of acquiring cancer, several immunological disorders, and several birth defects [13,14,64]. A recent investigation indicated that consumers indirectly swallowed a fungicide from the plastic used to wrap wheat bread, leading to the development of porphyria [1]. Table 3 presents different types of plastics and their environmental impact on human health risks.

#### 3.1.3. Microplastics’ Impact in Water Bodies and Oceans

**Figure 9:**
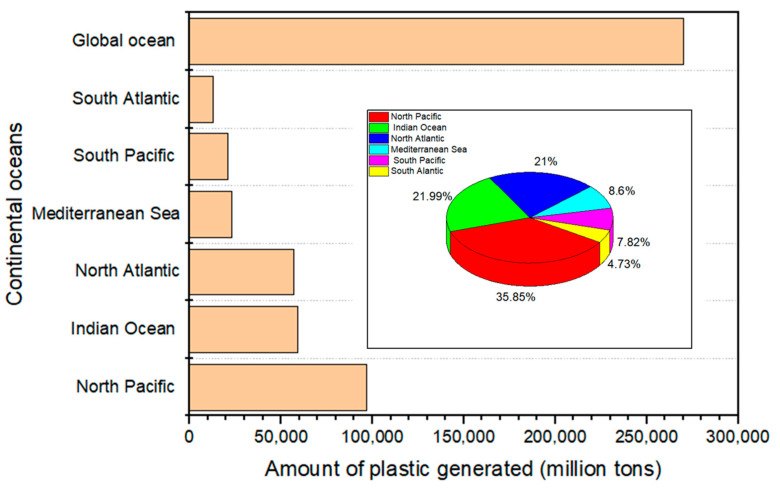
Distribution of plastic waste emission into the oceans adapted from [62].

The improper disposal of plastic garbage through illegal dumping and burning has health and environmental consequences. Plastic waste has a high menace of arriving at the ocean via wind or tidal transport or being transported to coastlines via interior waterways [22,55]. Substantial amounts of plastic debris and particulate plastics have been found globally in some freshwater bodies. A few field studies have been carried out in some parts of Africa. For example, studies have shown that populated urban areas influenced the distribution of microplastics in the Orange-Vaal River systems and Lake Victoria [35,43]. The Orange River system and Lake Victoria are important freshwater sources. Countries such as Kenya, Burundi, Tanzania, Rwanda, and Uganda depend on Lake Victoria for so many uses, including drinking water. However, contamination is unavoidable due to the anthropogenic activities resulting from plastic disposal [45]. Similarly, the land has been identified as the primary host for plastics, which are then transported to aerial and aquatic systems. Plastic contamination on the land was lower than observed on the Naivasha Lake in Kenya. Therefore, waterborne particulate plastics are persistent in certain locations. Particulate plastics are very buoyant, and atmospheric processes modulate their distribution in the air. Figure 9 shows how plastics are distributed worldwide in oceans such as the North Pacific (35.84%), Indian Ocean (21.99%), North Atlantic (21%), Mediterranean Sea (8.60%), South Pacific (7.82%), and South Atlantic (4.75%).

In Figure 9, we can see that the northern hemisphere ocean has the highest density of plastic waste. This was anticipated, given that most of the world’s coastal populations reside in the northern hemisphere [22,55]. The low coastal populations and plastic inputs in the southern hemisphere suggest that plastic pollution can be transferred between oceans and basins. This is because plastics are often buoyant and can easily float on the ocean’s surface to be transported by the major surface currents and wind pathways [52,59]. Consequently, sea surface currents and wind patterns significantly impact the distribution and accumulation of ocean plastics [62]. The pervasive presence of plastic waste accumulation in aquatic and agricultural environments poses a dire threat to global food security. As plastic infiltrates marine life, it faces ingestion or entanglement, while livestock suffer from consuming non-degradable plastic, resulting in a cascade of detrimental health consequences. Therefore, to develop solutions for plastic waste management in the oceans, extensive research needs to be conducted per the UN SDG 14.1 objective to reduce marine pollution, focusing on forms that originate from terrestrial sources, such as nutrient pollution and marine debris. Using the UN SDG Indicator 14.1.1 (measuring the coastal eutrophication and floating plastic detritus density) to assess this phenomenon, it is anticipated to make significant progress by 2025 [11,67].

### 3.2. The Role of Plastic Waste in Hindering the Achievement of UN SDGs in SSA

This section presents a summary of the primary barriers to enacting sustainable actions and strategies that are caused by the impact of plastic waste. At least 12 of the 17 UN SDGs were found to be directly or indirectly hindered by plastic pollution (Table 4). The plastics industry contends that plastic use will better the lives of people in developing countries where potable water is scarce, leading to high productivity of plastic sachets or bottled water. Consequently, implementing SDG 1 (no poverty) is difficult without adequate plastic reduction and monitoring targets [11,68]. The existence of (micro)plastics within agricultural soils and aquatic organisms presents potential hazards to human health, food security, and safety (SDG 2) via the consumption of consumables like bottled water, beer, salt, and bivalves [68,69]. Microplastics can accumulate in organs after being ingested (food) or inhaled (air), posing health risks (SDG 3). These impacts may cause cellular damage and inflammatory and immunological responses [70]. Microplastics are discharged into aquatic ecosystems through effluents and indirectly into agricultural soils through litter/landfill [71,72,73]. These circumstances present significant obstacles to establishing sustainable water and sanitation management practices for all individuals (SDG 6). Also, the non-renewable use of plastic waste for energy recovery contradicts the ambitions of attaining SDGs 7 and 9 [74]. Thus, plastic production and waste incineration emit 400 million tons of CO_2_ yearly, contributing to global climate change (SDG 7). This requires innovation (SDG 9) to go from a linear plastics economy involving fossil fuel-based production to sustainable bio-based plastics contributing to a circular economy [75]. In essence, high-income countries are more beneficiaries of the global circular economy, whereas low-income countries endure the full environmental consequences of waste made of plastics [11,76,77]. Poor-quality plastic waste and a lack of waste management infrastructure lead to unreported plastics being dumped, burned, or released into the environment [76,77]. These unsustainable scenarios worsen the disparity between developed and underdeveloped countries (SDG 10). In countries with poor waste management systems, plastic waste threatens their sanitation, urban infrastructure, and communities (SDG 11). Achieving SDGs 11 and 12 becomes very challenging because they require government and user cooperation to address plastic production, usage, and waste mismanagement [11,78].

Plastic polymers are made from fossil fuels, which increases the carbon footprint of greenhouse gas (GHG) emissions [75,79]. Thus, plastic waste and GHG emissions are interconnected, compromising the international community’s ability to achieve SDG 13. Plastic contamination threatens most marine and freshwater ecosystems globally. Herein, SDG 14 suggests the need to sustainably conserve and use the ocean, sea, and marine resources. However, no widely approved technique exists to measure this metric [67,73]. Furthermore, plastic contamination poses deleterious ecotoxicological effects on soil fauna (SDG 15). Despite the paucity of research on terrestrial soil microplastic pollution, recent interest in the detection, occurrence, characterization, and toxicology of microplastics in soil ecosystems has been substantial [11,69].

### 3.3. Addressing Plastic Waste with Adaptable Techniques Based on SSA Realities

The ability of plastic to undergo long-term transformation generates hazardous and toxic substances, which has a detrimental effect on the ecosystem and aquatic life. Generally, in SSA, open dumping, burying, burning, incineration, and landfilling are among the most conventional methods of managing plastic waste [58]. Therefore, adopting sustainable waste management practices is very important to prevent the harmful effects of hazardous or toxic waste, greenhouse gas emissions, water pollution, and air pollution impacts contributed by the tonnage of plastic waste that is annually generated. Therefore, without a sound environmental policy, Africa’s own and imported plastic waste will lead to a dramatic situation and require a global approach [58,80].

#### 3.3.1. Landfills and Recycling

**Figure 10:**
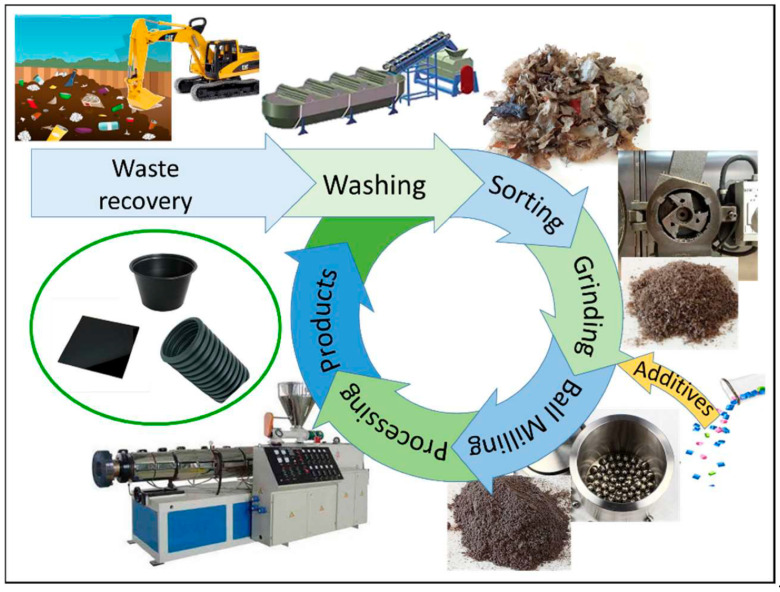
Schematic diagram of processing plastic waste by landfill recycling, adapted from [86].

Landfilling, recycling, and chemical and energy recovery are a few strategies being used in addressing the rising tide of plastic waste in SSA [52,54]. Landfills (Figure 10) are expanses of land constructed with various layers of soil and refuse for waste management [81]. In most developing countries, especially in SSA, plastic waste is not properly landfilled, and most of the time, openly incinerated in such landfills, contributing significantly to the carbon footprint and greenhouse gas emissions [58]. However, compared to alternative possibilities, landfilling municipal waste streams is less expensive, and is also not a sustainable method for managing plastic waste as it persists in landfills for up to 1500 years without deteriorating [8,28]. Also, without appropriate expertise in SSA to manage plastic waste and landfills. In South Africa, about 10% of plastic waste is openly incinerated; likewise, about 17% of plastic waste is openly incinerated in Ghana and 56% in Kenya, with all the associated negative consequences to the environment and the ecosystem [58].

On the other hand, recycling can help reduce the environmental impact of plastic waste; however, it is frequently more cost-effective to manufacture new plastic products from virgin materials than to recycle and reuse existing materials. It is especially important to conserve finite natural resources (fossil fuels), as the plastic manufacturing process consumes nearly 8% of the total quantity of oil produced worldwide [82,83]. The practice of reusing plastic materials rather than disposing of them after their contents have been consumed is commendable in SSA [26].

In a portion of the SSA region, the direct utilization of uncontaminated waste plastics to produce new products without compromising their properties has yet to be explored [15,28]. To further process recyclable materials, several of the facilities designed in SSA, such as buy-back centers, drop-off centers, and roadside collecting centers, have significant drawbacks [13,84,85]. These disadvantages include the following: (i) Losing secondary materials during the collection, processing, and subsequent processing. This is because recycling cannot guarantee complete resource recovery from refuse materials [52]. (ii) Inadequate facilities are impeding the recycling of different waste materials [22]. (iii) Polluted recyclables pose some issues, such as materials being extremely difficult to separate, and so can compromise the product quality [22,62]. Moreover, most plastics are non-biodegradable, making recycling difficult [17,52].

#### 3.3.2. Photochemical Degradation

**Figure 11:**
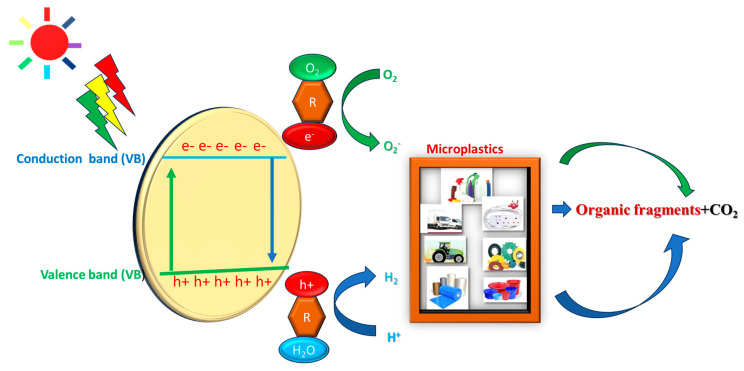
Schematic diagram of the photodegradation mechanism of micro-and macro-plastic waste.

Photochemical degradation (Figure 11) involves using a light source in depolymerization and decomposition reactions to lower the molecular weight of the plastics [87,88]. Initiation, propagation, and termination are the three basic phases of the plastic photodegradation mechanism [80]. Free radicals are produced when light or heat disrupts the chemical bonds in polymer chains during the initiation phase [88]. For polymers to absorb light energy, unsaturated chromophoric groups must be present. The photo-oxidation of plastic surfaces during abiotic degradation increases the hydrophilicity of the polymer and facilitates the formation of microbial biofilms [87]. Consequently, the surface area of the produced molecules increases, rendering them more susceptible to fragmentation. The photodegradation of plastic waste involves three stages: (i) quick CO_2_ release and O_2_ absorption to the equilibrium phase, (ii) a drop in the degradation rate, and (iii) a rapid deterioration of the surface structure [89,90]. Scott [91] reported that polystyrene waste (PSW), which could be expanded polystyrene or polystyrene packaging, was recycled via photochemical reaction to generate organic–inorganic hybrid materials (nanocomposites).

#### 3.3.3. Thermal Degradation

**Figure 12:**
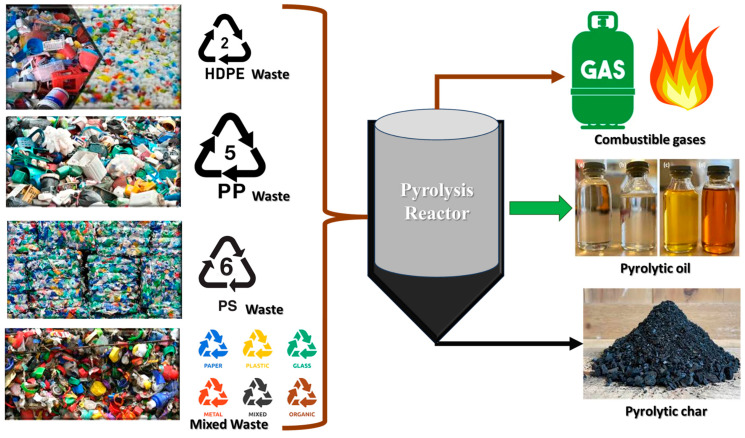
Schematic diagram of thermal pyrolysis of plastic waste into pyrolytic oil, chair, and gases.

Thermoplastic polymers can undergo thermal degradation (Figure 12) during processing at high temperatures (T > T_m_), thereby transforming the polymer from a solid to a liquid phase [92]. The degradation reaction involves trace amounts of water hydrolysis, oxidative degradation, cis-elimination, and inter- and intramolecular transesterification reactions [87,88]. CO, CO_2_, acetaldehyde, and methyl ketene generation occurs because of inter- and intramolecular ester exchange, cis-elimination, and radical and coordinated non-radical reactions [92,93]. Depending on the plastic polymer’s nature and properties, plastic’s thermal degradation can occur at high temperatures, typically greater than 100 °C [93]. Antioxidants in plastic manufacture prevent low-temperature thermal oxidation [94].

**Figure 13:**
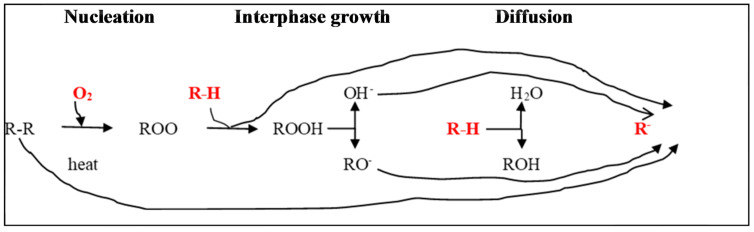
Schematic representation of the thermal degradation mechanism of plastic waste.

Stress and other reactive chemicals, like ozone, accelerate heat oxidation-induced degradation. Polybutadiene (PBD) and polyvinyl chloride (PVC) are thermally degradable polymers due to their chemical makeup [88,91]. In contrast, polymers with strong backbone bonds, like polysulfone, polyether ketone, and polysiloxanes, are heat-resistant [93]. Figure 13 shows the mechanism and factors that affect thermal degradation. However, the solid-state conversion reactions (nucleation, interphase growth, and diffusion) are prone to being restricted by the simultaneous occurrences of complex layers (R-H) [5,87,88]. Moreover, the impact of thermal degradation under conventional environmental conditions is considered insignificant, especially in icy marine habitats and energy regions [8,22].

#### 3.3.4. Limitations of the Thermal and Chemical Degradation of Plastic Waste

Plastic waste has supplanted other materials, such as wood, metal, and glass, in applications where plastics dominate. However, their resistance to degradation makes plastic waste very challenging or impossible for natural absorption and degradation [13,22]. The fragmentation of plastic waste can produce brittle materials or plastic waste of various sizes. These sizes can be classified as nanoplastics (0.1 m), microplastics (1–4.75 mm), mesoplastics (4.76–200 mm), or macroplastics (>200 mm) [10,93]. The exceptional malleability of the categorized plastics explains the increase in yearly production and waste pollution. Plastics can degrade via a variety of methods, including thermal, chemical, photochemical, and biological processes [8,18,91,92]. However, these processes can be affected by external factors such as temperature, plastic weight, mechanical stability, intramolecular tensions between the chains, and polymer chain length [8,18]. In addition, the molecular weight of a plastic might influence its decomposition rate. In this theory, polymers with a high molecular weight and a low relative surface area degrade more slowly [8,92]. The chain length and bond composition affect a polymer’s susceptibility to abiotic and biotic breakdown [8,90]. Hence, plastic’s physicochemical properties play a crucial role in its deterioration. Additionally, hydrophobicity frequently affects the efficiency of plastic polymer degradation, with the degradation rate increasing as hydrophilicity rises. Plastic polymers like PET contain heteroatoms, organic molecules that decompose into carbon dioxide and water [93,95]. The crystallinity of the polymer also influences the degradation rate of polymeric polymers; compared to amorphous polymers, the more crystalline the polymeric structure, the greater the need for water and oxygen to commence the breakdown process. The amorphous parts of a polymer are also thought to be more susceptible to thermal oxidation [91]. On that note, microbial biodegradation could be viewed as a viable, environmentally acceptable approach to combating plastic pollution in SSA. In this context, microbial and bioremediation are furthermore discussed as prospective techniques adaptable for mitigating plastic waste in SSA.

## 4. Emerging Microbial and Bioremediation Management of Plastic Waste Technologies

**Figure 14:**
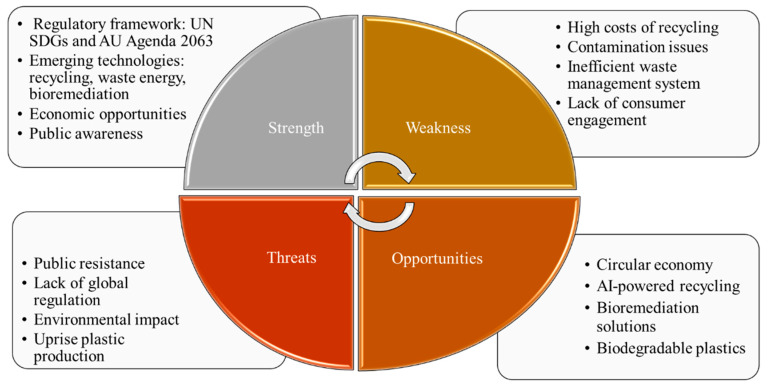
Schematic diagram of strengths, weaknesses, opportunities, and threats (SWOT) analysis of plastic waste management via bioremediation.

In SSA, it is essential to improve plastic waste management strategies through a better collection system, a ban on improper landfilling, the reuse of plastic products, and the improvement of plastic recycling by the local plastics industry [58]. On the contrary, physicochemical management techniques of plastic waste, such as landfills and incineration, were more prevalent in the SSA. However, the rising plastic production demand and lack of global cooperation pose significant environmental threats. The challenges associated with plastic waste remain inefficient waste management systems with high recycling costs, contamination issues, and public resistance. Hence, the strengths, weaknesses, opportunities, and threats (SWOT) analysis of plastic waste management was carried out, as shown in Figure 14. It was found that the rising awareness, technological advancements, and regulatory frameworks (UN SDGs and African Union Agenda 2063) create opportunities for bioremediation as a sustainable solution for plastic waste management.

### 4.1. Microbial Biodegradation

Microbial biodegradation involves anaerobic conversion of organic molecules into biogas and residual biomass [45,85]. Herein, carbon-rich plastics, such as polyethylene sulphide (PES), polyvinyl chloride (PVC), polyamide (PA), and polyurethane (PU), can be utilized as carbon sources for biodegradation [96]. However, plastics are poor growth media for microorganisms. Consequently, only a small percentage of plastic waste can be biodegraded [27]. The polymer’s molecular weight impacts the rate of degradation and solubility. Therefore, microorganisms can accelerate the oxidation–reduction reaction to break down the chemical bonds in the plastic polymer [81,90,97]. In addition to the surface area, functional groups, molecular weight, hydrophilicity, hydrophobicity, melting temperature, chemical structure, and crystallinity can impact the biodegradability of a plastic [8,25,87]. Introducing microbes to plastics accelerates its hydrolytic cleavage into oligomers, dimers, and monomers, which can then mineralize it into CO_2_ and H_2_O [55,98]. Polymers with amorphous domains are consequently more susceptible to microbial degradation [45]. These microbes produce extracellular and intracellular laccases that can catalyze the oxidation of certain inorganic ions as well as ortho and para diphenols, aminophenols, polyphenols, polyamines, lignins, and polyamines [20,45,99]. Furthermore, the hydrophobic properties of plastic polymers limit microbial activity by preventing water absorption. However, this can be addressed by creating biofilms [100,101]. Tkachuk and Zelena [102] reported that *Bacillus mycoides* and *Penicillium frequentans* can form a biofilm to facilitate plastic decomposition.

### 4.2. Mechanism of Microbial Degradation of Plastics

**Figure 15:**
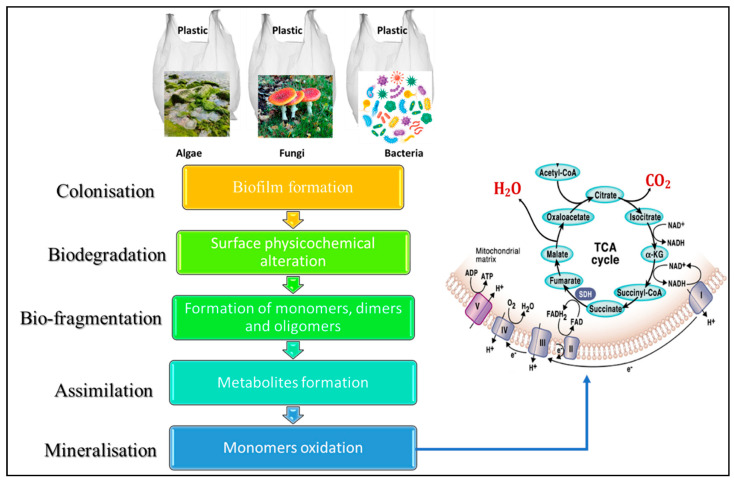
Schematic representation of microbial degradation of plastic adapted from Jaiswal et al. [105].

Colonization, biodeterioration, fragmentation, assimilation, and mineralization are the microbial mechanisms that cause plastic waste to degrade [96,100,103]. However, microorganisms (algae, bacteria, and fungi) can infiltrate polymer materials and cause an increase in holes and cracks, which can lead to physical biodeterioration. The production of extracellular molecules by microbes that change the hydrophobic and hydrophilic phases can also increase the rate at which microbes penetrate a surface [100]. These extracellular compounds speed up the build-up of contaminants, which speeds up microbial development and the pace of biodeterioration [45,85]. Figure 15 presents the generalized scheme for microbial degradation with four essential steps. The colonization of microbial species on the plastic surface is the initial stage of the microbial degradation mechanism. Biofilm development by one species or a microbial consortium leads to plastic polymer surface degradation [81,90,97]. This stage can be examined with electron, photonic, or polarization microscopy. As demonstrated in Figure 15, the adhesion of microorganisms to plastic surfaces can be facilitated by forming different polysaccharides and/or hydrophobic proteins (cysteine-rich proteins). These chemicals permeate the polymer, modify its pore size, and alter the morphology of the plastic’s surface [90,97,104].

A physicochemical transformation occurs at the biodegradation phase (Figure 15), and the durability and resistance of plastics decrease as their surface area grows. This demonstrates the importance of extracellular enzymes and secretions in biodegradation. Also, *chemoorganotrophic* bacteria, such as *Nitrobacter* spp. and *Thiobacillus* spp., can excrete organic acids like oxalic, gluconic, citric, and glyoxylic acids. These organic acids are essential for the process completion by effectively preventing biodeterioration and fixing cations than mineral acids [106]. On this basis, the pH of the medium plays a major role in the metabolic processes. As shown in Figure 15, at the bio fragmentation stage, a lytic process is necessary for decomposing polymers into monomers, dimers, and/or oligomers. The microorganisms cleave to the plastic polymers by secreting oxidoreductases, hydrolases, and free radicals [100,103]. On the other hand, free radical oxidation aims to boost the molecule’s polarity by the addition and/or production of a hydroxyl function and carbonyl or carboxyl group. This enhances the hygroscopic characteristics to promote microbial neutrality [45,101]. Moreover, some enzymes can catalyze oxidation processes, generating free radicals that produce oxidative stress, leading to chain reactions and triggering polymeric structure conversion and degradation. Moreover, the secondary metabolites produced by assimilation can be transmitted beyond the microbial cell and utilized by other microorganisms that undergo additional degradation. Nevertheless, the degradation of metabolites, both primary and secondary, releases oxidized products such as CO_2_, N_2_, CH_4_, and H_2_O. This can occur under anaerobic, aerobic, or fermentation pathways to produce energy-carrying molecules like adenosine triphosphate (ATP) [100,105]. Biodegradable plastics are called bioplastics. This indicates that these polymers can be created using either fossil fuels, biomass, or other renewable resources.

#### 4.2.1. Role of Bacteria and Fungi

Fungi or bacteria use plastics as a carbon and energy source to break the main chain cleaves, forming oligomers, dimers, or monomers [107,108]. Mycelia penetrate the polymeric material’s surface to disintegrate as many substrates as possible. These monomers are subsequently taken up by fungi to be assimilated or mineralized by their intracellular enzymatic machinery [105,107]. Fungi generate and secrete more enzymes than bacteria. According to [107], white- and brown-rot fungi have frequently been reported as effective degraders of polymeric compounds. Fungi can degrade plastics due to their enzymatic system, which excretes manganese peroxides, lignin peroxidase, versatile peroxidase, and laccase [107,109,110,111]. Polymeric materials have similar physical qualities to lignin, such as hydrophobicity and chemical structures (ether bonds, non-phenolic aromatic rings, carbon skeleton, etc.) that are oxidized during lignin degradation [107,109]. Physical and chemical similarities allow lignin-modifying enzymes to degrade plastic polymers (e.g., PE and PP). Herein, the resulting fragments must be thoroughly utilized by the microbes to avoid environmental and health repercussions. Table 5 presents some examples of fungi and bacteria used for plastic degradation.

#### 4.2.2. Role of Algae in Microbial Degradation

Filamentous algae can colonize plastic wastes due to available sunlight, nutrients, and water [113,114]. Bacterial and fungal systems may be biological pollutants due to endotoxins and the need for a rich carbon source. Several non-hazardous algae species (*Bacillariophyceae*, *Chlorophyceae*, and *Cyanophyceae*) colonized on polyethylene-formed biofilms in contaminated ponds, lakes, and wastewaters. Sarmah and Rout [113] demonstrated that *Phormidium lucidum* and *Oscillatoria subbrevis* (non-toxic freshwater cyanobacteria) can colonize polyethylene (PE) surfaces and destroy LDPE without pretreatment. The diatom *Phaeodactylum tricornutum* was employed as a microbial factory by Hempel et al. [117] to produce an engineered PETase (polyethylene terephthalate glycol). Khoironi et al. [118] discovered that algal colonization, such as *Spirulina* sp., reduces the tensile strength of PET and PP by 0.9939 and 0.1977 MPa/day, respectively. Algae adhere to plastic and break it down by producing exopolysaccharides and ligninolytic enzymes. Genetically engineered microalgae can produce polymer breakdown enzymes. *Chlamydomonas reinhardtii* (green microalgae) modified genetically was tested against PET [113,118,119]. Moreover, the prospects of algae coupled with nanoparticles are gaining attention to be utilized to produce a biodegradable PET solution [113,120]. However, the mechanism and efficiency of algal plastic degradation require further study.

### 4.3. Bioremediation of Plastics

The amassing of plastic waste is a huge environmental problem. Bioremediation using biological systems is gaining a reputation in pollution management, especially for plastic waste [110,121]. Shilpa et al. [45] reported bioremediation as a natural, cost-effective way for waste to deteriorate compared to other physiochemical techniques. Plastic waste can be mineralized completely by the bioremediation process. This is accomplished through several enzymes and their production pathways, which generally do not exist in a single strain but are easily produced by a microbial consortium [110,122]. For instance, a mixed culture of *Lysinibacillus xylanilyticus* and *Aspergillus niger* isolated from the landfill soils of Tehran was used for the degradation of low-density polyethylene (LDPE) [110]. The main mechanisms used by microbes to improve, foresee, and assess cellular function, gene products, and survival tactics under stressful environments are mostly revealed by biological systems [18,110].

Additionally, the microbe’s genome, RNA, protein, and other metabolites are produced, facilitating microbial cell–cell interactions in communities. This enhances the possibility of optimizing the target microbial consortiums for functional designing of the genes, metabolic pathways, and metabolic networks [114,123]. Table 6 presents bioremediation technologies for the degradation of plastic waste. However, their intrinsic properties hinder the bioremediation of plastics, which prevents their decomposition into monomers [110,122]. Microbial enzyme systems, on the other hand, are also ineffective against non-hydrolyzable synthetic plastics [100,123], whereas genetically engineered bacteria can break down a wider range of contaminants with microbe-based bioremediation [115,124].

### 4.4. Genetic Modification/Green Plastics

The adaptability and implementation of biodegradation techniques with microorganisms to digest plastics, as well as modification into green plastics, can have a sustainable impact on SSA. Biodegradation of plastic waste interacts symbiotically with heterotrophic microorganisms such as yeast, bacteria, and fungi, resulting in the exchange of nutrients and metabolites that increase bioremediation. This makes the bioremediation of plastic more effective with a tailored microbial community through enzymatic activity. Nevertheless, algal biodegradation, a recent up-cycling method, is expected to be better than bacterial or fungal biodegradation [113,119]. Algal biodegradation requires no special pretreatment or a carbon-rich source. The oxidative stress caused by free-radical-producing algae is attributed to increasing the polarity of plastic polymers, which in turn facilitates their biodegradation [114].

Genetically modifying microorganisms to degrade recalcitrant plastics requires characterizing genes that produce degrading enzymes and their control [115,124]. Analyzing metabolites produced during degradation determines the efficiency of the acting microbial candidate. However, a screening program for such organisms and enzymes is expected, and it will necessitate more highly standardized criteria for evaluating their degradative capacity [5]. To be considered green or environmentally friendly, the impact of a material’s incursion or degradation in a particular ecosystem must be either neutral (have no net effect) or positive (energy-efficient, readily recyclable, or reusable, etc.). We consider “green plastics” biodegradable, biocompatible, and environmentally neutral or beneficial. Thus, “green plastic” is a polymeric material that is comparable to or better than conventional polymers and has a lower environmental impact.

Furthermore, using nanotechnology to produce plastics may address the problem of plastic biodegradation and adjust the frequency of biodegradation [127]. Therefore, to preserve human health and the environment, more research is needed on the negative consequences of accumulated plastic waste in marine and soil habitats and their effects on fauna and flora. In addition, polymer-degrading microorganism mechanisms and environmental factors that contribute to controlling plastic degradation require thorough investigation. Therefore, the adaptability and implementation of the biodegradation technique present cost-effective and sustainable options for degrading plastic waste via natural mediums.

### 4.5. Microplastic as Vector for Antibiotic-Resistant Bacteria and Genes (ARB and ARGs)

The presence of antibiotic-resistant bacteria and genes (ARB and ARGs) on microplastics (MPs) in a variety of settings, including air, aquatic and terrestrial habitats, and wastewater treatment facilities (WWTPs), has been investigated by several researchers [116,128,129,130,131]. WWTPs were found to be the hotspots for ARGs and MPs [129,132] In terrestrial and marine cultures, micro (nano)plastics and resistant genes/multidrug-resistant strains have frequently been discovered alone or in combination as new contaminants [116,130,133]. However, MPs carrying ARGs encourage genetic contamination, which travels from the ocean circulation to the shore, then via the food web from the enriched marine life there to aquaculture, where it eventually reaches living organisms and causes unfathomable harm [131,134,135]. Beyond the environment, microplastics have also become a breeding environment for the evolution and transmission of metals and ARGs at the supramolecular level. Table 7 presents recent research highlighting the fate of microplastics as vectors of antibiotic-resistant bacteria and genes (ARB and ARGs). The MPs build up in the food chain and plant uptake is a grave concern that requires research attention. Because of its effects on carbon sequestration and biogeochemical cycling, it poses a significant threat to the ecosystem. Consequently, MPs have become a hotspot for transferring drug-resistant pathogenic genes and are disease vectors in water and other pollutants [129,132]. Moreover, MPs and ARGs work together to spread genes and pollute the environment, putting human health and aquaculture at risk [128,130]. Therefore, future investigations into the effects of microorganism habitats on MPs and the exchange of genes between species are necessary for SSA to combat plastic pollution. Moreover, the type of polymer, ARG subtype, environment, and incubation duration are the main factors that influence the enrichment of ARGs in microcosm and microplastic studies on WWTPs and landfill leachate [131,132,135,136,137].

## 5. Challenges and Future Research on Plastic Waste Management in SSA

### 5.1. The Lack of Policies and Inconsistencies in Data Availability

Reducing plastic pollution requires understanding the socio-economic, physicochemical, and environmental factors that affect plastic waste from the production, distribution, usage, and disposal routes. However, in SSA, there is limited information or data available to support plastic mitigation and technological advancement. In contrast, plastic pollution in SSA is escalating with serious health and management challenges [22,57]. However, SSA countries lack environmental pollution information and policy implementation knowledge. Therefore, addressing waste made of plastic requires creating laws, legislation, and initiatives that protect the environment and public health. In this context, the authors believe sustainable solutions with improved waste management systems are required. And there is an urgent need for the enactment of education on sustainable plastic use and zoning policies. Although there are 17 SDGs, 12 (Table 2) of them were identified to be undermined by the emission of plastic waste into the environment. Also, governmental and commercial partnerships can stimulate a policy for using renewable materials for plastic production [11,78]. In addition, public education can be encouraged to reduce the influx of plastic usage, which has become part of our daily activities [7,18,25,28]. Even though it is not feasible to eliminate all plastics from society, prospective alternatives can play an important role in replacing conventional plastic-oriented waste production [18].

Microplastic pollution intervention policies in Africa vary greatly due to the weight countries place on policy drivers (e.g., reducing pollution, protecting human and environmental health, promoting environmental leadership, and creating green jobs) versus the socioeconomic cost of reducing plastic usage [7]. Herein, plastic is one of the most pervasive materials in the world, and its value chain and environmental sustainability require research attention. For instance, researchers can evaluate the health impacts of microplastic waste, create novel techniques to reduce microplastic pollution, and provide information to the government to help it enact legislation that would motivate all stakeholders in the value chain to achieve their plastic waste reduction objectives [7,140]. In addition, we propose a revision of tariffs and levies on reclaimed or recycled plastic goods and materials across the entire value chain to make them economically viable and competitive with virgin resin products, thereby encouraging businesses to get involved with the remediation of the environment. It has been posited that a collaborative effort between policymakers, governments, and the general populace is imperative in establishing a viable circular economy for plastic. Therefore, all stakeholders must cooperate to mitigate plastic pollution effectively.

### 5.2. Valorisation of Plastic Waste into Fuels

With the energy crisis in SSA, the valorization of plastic into biodiesel and syngas comes with both economic value and sustainability benefits. Recycling plastic waste to create biofuels, bioplastics, biofertilizers, and cementitious composites for building are all benefits that are rapidly gaining popularity as a viable route to ending plastic waste [141,142]. In this scenario, anaerobic digestion of plastic waste in the presence of methanogens will potentially produce methane gas (biofuel) [82,143]. Similarly, employing plastics as a source for the growth in algal biomass can result in the exploitation of its potential benefits, including the generation of biofertilizers and adsorbents [56,140]. To reduce plastic pollution worldwide, plastic waste must be converted into fuel. Some of these possible solutions for valorizing plastic waste range from primary routes of direct recycling to quaternary routes of valorizing plastics to energy. The proposed process routes include (i) gasification of plastic waste, which deals with the production of gaseous streams for energy, (ii) pyrolysis to produce H_2_, and (iii) integration of plastic-derived products or plastics into refinery units [56,87,140].

### 5.3. Future of AI-Powered Plastic Waste Management

**Figure 16:**
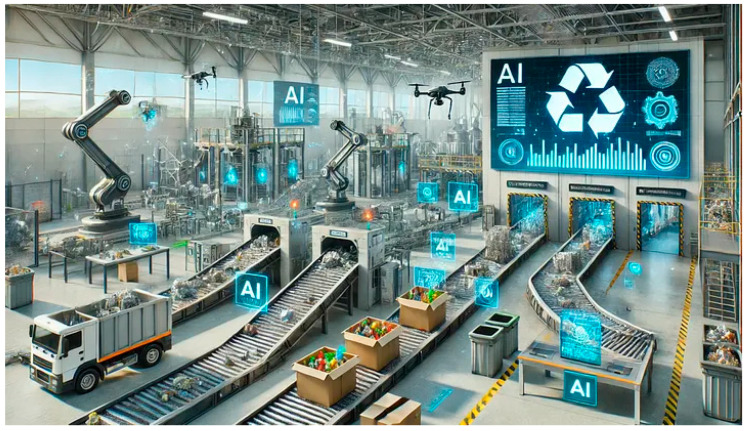
Schematic diagram of AI-powered automated recyclable system (OpenAI-ChatGPT, Generative Image accessed on 22 December 2024).

In the face of growing waste challenges, AI-powered solutions (Figure 16) present a critical step to transforming waste management. Subsequently, with the complexity of sorting recyclable plastic waste for proper disposal, it is important to keep pace with rapid change for technological advancement in SSA. Hence, the integration of AI-powered image recognition with barcodes into waste management systems presents a revolutionary potential for SSA industries and municipalities for more efficient and environmentally sustainable practices. By leveraging advanced machine learning algorithms, image labeling, and optical character recognition in recycling processes, AI-powered systems are more essential. Some of the AI-powered system benefits extend beyond the operational efficiency of automated waste sorting, monitoring, and hazardous waste detection for safe disposal with a streamlined, improved recycling rate. Sustainable future practices of plastic waste have more resource recovery potential with continuous production, with real-time monitoring for a sustainable circular economy.

The recommendations for researchers, policymakers and Non-Governmental Organizations are summarized as follows:

- AI-powered image (Figure 16) recognition integrated with smart city initiatives and Internet of Things (IoT) devices, adaptation, and implementation will create more efficient data-driven systems with low operational costs and environmental impact contributing to a more scalable efficient and a circular economy of plastic waste.
- Sustainable solutions with improved waste management systems and urgent education on sustainable plastic use and zoning policies are required.
- Revision of tariffs and levies on reclaimed or recycled plastic goods and materials across the entire value chain to make them economically viable and competitive with virgin resin products, thereby encouraging businesses to get involved with the remediation of the environment.
- The use of nanotechnology in the production of plastics may address the problem of plastic biodegradation and adjust the frequency of biodegradation. In addition, polymer-degrading microorganism mechanisms and environmental factors that contribute to controlling plastic degradation require thorough investigation.
- To reduce plastic pollution worldwide, plastic waste must be converted into fuel; this can be carried out via valorization. Some of the possible solutions for the valorization of plastic waste range from primary routes of direct recycling to quaternary routes of valorizing plastics to energy.
- Evidently (Table 7), MPs can serve as vectors for many antibiotic-resistant bacteria and genes (ARB and ARGs).

### 5.4. Life Cycle Assessment and Upcycling

Recent studies using life cycle assessment (LCA) provided critical insights into the environmental performance of different types of plastic waste management frameworks. Ideally, mechanical recycling due to the avoidable use of virgin plastic has a low environmental impact, especially on global warming and energy use. Though the practical application of recycling remains a challenge, it enhances plastics’ sustainability. As shown in Table 8, a summary of studies conducted on LCA plastic waste management is highlighted [144].

In contrast, incineration with energy recovery performs better than landfilling in terms of energy efficiency but results in significantly higher GHG emissions. Pyrolysis and chemical recycling are emerging as promising technologies, particularly for mixed or contaminated plastic streams. While their environmental impacts are currently higher due to energy demands, improvements in process efficiency and renewable energy integration could enhance their sustainability [145,146,147,148]. However, a hybridized approach of recycling with chemical or thermal processes also enhances valuable resources’ recovery while minimizing carbon footprint and global warming [145,146]. It also emphasizes the importance of local infrastructure, energy mix, and policy that can enhance the effective mitigation of plastic waste. This alludes to the fact that LCA supports the petrification of recycling over incineration, which has been the most common approach in SSA [145,146].

**Table 8:** Summary of life cycle assessment and recycling on plastic waste management.

Study	Focus	Plastic Waste/Process Type	Environmental Impact	Key Findings
[149]	A comprehensive critical review of Life Cycle Assessment applied to thermoplastic polymers for mechanical and electronic engineering	Thermoplastics	GHG emission, energy usage, fossil fuels, climate change, and global warming	Provision for sustainable manufacturing and application of thermoplastic
[150]	High-resolution life cycle carbon footprint analysis for footwear products underpinned by on-site measured data	Mixed plastics	High carbon footprint, energy-intensive	>90% carbon footprint emitted from the feedstock used.
[146]	Techno-economic and life-cycle assessment for syngas production using sustainable plasma-assisted methane reforming technologies	Mixed plastic, recycling, and reforming process for syngas production	Carbon footprint, energy intensity, and eutrophication potential	Impacts of plastic on UN SDGs (#7, #9, and #13) were explored. Promoting material recyclability contributed to sustainable industrial practice (UN SDG#12)
[151]	Integrating waste thermal conversion and lifecycle analysis for sustainable energy production: Reflecting upon environmental and economic impacts	Mixed plastic to energy mix different production routes	Environmental impact, cost, and energy were considered	Incineration has shown higher impacts than gasification schemes for global warming potential, marine and human toxicity, and lower in eutrophication, abiotic depletion, and terrestrial toxicity
[152]	Exploring Temporal, Regional and Stakeholder Dimensions of Carbon Fiber-Reinforced Polymers (Cfrps) Recycling: A Life Cycle Assessment Case Study	Mixed plastic with pyrolysis and solvolysis as two recycling technologies	Supports recycling strategies within a circular economy.	Pyrolysis generally achieves lower environmental burdens at the process level
[145]	Towards Sustainable Municipal Solid Waste Management: An SDG-Based Sustainability Assessment Methodology for Innovations in sub-Saharan Africa	Mixed plastics and municipal solid waste	Energy, cost, and environmental impact	Promote sustainable practices while fostering sustainable development in SSA
[148]	Plastic Waste Management: A Review of Existing Life Cycle Assessment Studies	Mixed plastic management	Six parameters have been considered to analyze research progress in the fields regarding LCA, i.e., goals and scope, functional units, impact assessment categories, system boundaries, geographical context, and uncertainty analysis.	Recycling was recommended as an effective tool
[147]	Life cycle assessment of plastic waste and energy recovery	Mixed plastic	environmental impact	Conversion factors for the metrics harmonization were provided. Landfills, disposal, and treatment were highlighted

## 6. Conclusions

In conclusion, based on the literature consulted, plastics were found as the most indispensable materials in every facet of life, including the automotive, packaging, health, and manufacturing sectors. However, global plastic waste production has been found to pose both environmental and health-related problems, which underline the UN SDGs. This review’s findings highlighted major concerns about plastic waste, its usage, and its degradation approach via burning and incinerating in the SSA region. Besides the physicochemical approaches, the development of bioremediation and microbial degradation of plastic waste was also examined. It elucidated the conceptual and applicability role of fungi, bacteria, and algae in the biodegradation of plastic waste. Also, pyrolysis and bioremediation via anaerobic digestion were proposed as options to explore in generating syngas from plastic wastes. In the SSA region, there is still a lack of viable, environmentally acceptable, and cost-effective plastic degradation processes for the green energy economy; therefore, it must be given attention.

Recycling is more prevalent in most developed nations; similarly, in some SSA regions, the same is observed. The review recommends implementing plastic waste recycling regulations to be observed by all nations. Consequently, microbial and bioremediation research is required to develop innovative ways to reduce the production of plastic waste. Moreover, modification of plastic products into green/biobased plastics with significant degradation and recyclability potential can reduce the environmental footprint of plastic waste. Furthermore, to ensure sustainable consumption, production, and a reduction in plastic waste as a means of meeting the UN SDGs, collective effort is required by the international community. Thus, there is a need to develop circular economies where end-of-life plastics are valued rather than becoming waste and dramatically increase domestic recycling rates without relying on the global plastic waste trade. Furthermore, risk assessment for internalized plastics is hindered by the absence of exposure thresholds and ambiguities in toxicological risks. A holistic approach using quality-assured techniques like life cycle assessment (LCA) is recommended to evaluate microplastics in drinking water and environmental matrices, along with their inhibition on microbial community shift.

## Figures and Tables

**Table 1:** Considerable research themes and areas associated with cluster network keywords.

Research Theme	Ref	Research Area	Publication Citations	Remarks
Mineralisation and metabolic techniques (Cluster 1)	[31]	Engineering; Environmental Sciences and Ecology	27	Biodegradability with 30 mg/g bacterial incubation for 10 days
	[32]	Biotechnology and Applied Microbiology; Environmental Sciences and Ecology	100	Biodegradation processes such as biochemical and aerobic degradation are needed for a sustainable bioeconomy. Explore the potential of microorganisms (bacteria and fungi) and biochemical for plastic waste degradation
	[33]	Environmental Sciences and Ecology	127	SSA market hub of plastic and drawbacks such as high energy and limited technology to mitigate its waste produced
	[34]	Environmental Sciences and Ecology	184	Biodegradation of plastic waste via microbes and algae
	[35]	Engineering; Environmental Sciences and Ecology	40	Bacterial degradation of plastic with strain NyZ600
	[36]	Biochemistry and Molecular Biology; Biotechnology and Applied Microbiology	67	Bioremediation and genetic engineering strategies for the degradation of plastic waste
	[37]	Environmental Sciences and Ecology	244	Biodegradation of polymers and microbial engineering strategies
	[38]	Biotechnology and Applied Microbiology; Environmental Sciences and Ecology	127	Exploring the potential of biotechnology such as enzymatic engineering and plastic bioconversion into valuable products
	[39]	Biotechnology and Applied Microbiology; Chemistry; Engineering	79	Explore the potential of genes, enzymes, and interaction between microbes and plastic as a substrate to enhance biotechnological processes
Micro-and bio-based degradation mechanism of plastic waste (Cluster 2)	[40]	Biotechnology and Applied Microbiology	111	Prospects of microbial degradation of plastic waste and its challenges
	[41]	Chemistry; Science and Technology—Other Topics	135	Bio and microbe-based biotechnologies for the alleviating of environmental challenges and biodegradation of plastic waste
	[42]	Engineering; Environmental Sciences and Ecology	34	Biodegradation of 30 days substantiated the efficacy of enzymatic degradation
	[43]	Biochemistry and Molecular Biology; Pharmacology and Pharmacy	137	Microbial enzymatic degradation is suitable from a bioremediation point of view as no waste accumulation occurs.
	[44]	Microbiology	233	Microbial and enzymatic degradation is a promising strategy for depolymerization of waste petro-plastics into polymer monomers
	[45]	Engineering; Environmental Sciences and Ecology	201	Prospects of microbial species, genes, biochemical and enzymatic pathways for plastic biodegradation
	[46]	Engineering	92	Potential of degrading polymers with microalgae
Challenges and prospects of bioremediation technologies (Cluster 3)	[47]	Environmental Sciences and Ecology; Geology	119	Microbial remediation and bioleaching mechanism of plastic via the transformation, biomineralization, and bioaccumulation
	[48]	Environmental Sciences and Ecology; Public, Environmental and Occupational Health	202	Exploring recycling, depositing in landfill, incineration, microbial degradation, and conversion of plastic
	[49]	Science and Technology—Other Topics; Engineering; Environmental Sciences and Ecology	104	Challenges and prospects associated with plastic degradation
	[50]	Environmental Sciences and Ecology	178	Microbial degradation factors and enzymatic mechanism

**Table 2:** Plastic waste generation across SSA regions.

Region	Country	Estimated Annual Plastic Waste Generated (Metric Tonnes)	Key Observation	References
North Africa	Egypt	3,037,675	Largest producer in Africa with a significant contributor to the Mediterranean pollution	[22,58]
	Algeria	2,092,007	High consumption of plastic bags with substantial mismanagement and pollutants in the Mediterranean Sea	[26]
Southern Africa	South Africa	2,425,323	Advanced recycling infrastructure yet significant plastic leakage and contributor to the Indian Ocean	[26,58]
	Mozambique	700,000	High plastic leakage into marine environments (Indian Ocean) and limited domestic production	[26]
East Africa	Kenya	880,000	Implemented plastic bag bans, faced challenges with waste collection. Pollutants in the Indian Ocean	[22,58]
	Ethiopia	900,000	Limited waste management systems and increasing plastic consumption. Contribute to Indian Ocean pollution	[26,58]
West Africa	Nigeria	2,459,502	Rapid urbanization, challenges with informal recycling sectors. Major pollutant of the Atlantic Ocean	[58]
	Ghana	1,100,000	Emerging recycling initiative and infrastructure development; major contributor to Atlantic Ocean pollution	[58]

**Table 3:** The use of plastic and the associated health and environmental consequences.

Plastic Types	Uses	Effects	Refs
Polyethylene terephthalate (PET)	Food and beverage containers	Carcinogenesis, vomiting, and darkness	[1]
High-density polyethylene (HDPE)	Milk jugs, detergent and juice bottles, toiletries containers	Stomach ulcers, not biodegradable	[1,6]
Polyvinyl chloride (PVC)	Food wrap, bottles for cooking oil, shower curtains, inflammable mattresses, common plumbing pipes	Interfere with hormonal development	[1]
Low-density polyethylene (LDPE)	Bottles and bread wraps	Non-hazardous. No health effects. Recyclable	[6]
Polypropylene (PP)	Food containers, yogurt cups, medicine and ketchup bottles, kitchenware	Possibility of chemical leaching	
Polystyrene (PS)	Packaging cups, takeaway materials	Takes 1000 years to decades	[56]
Polycarbonate and others	Baby and water bottles, sports equipment, medical and dental devices, CDs and DVDs, computers	Obesity, cancer, endocrine problems in fetuses and children	[13,14].
Microplastics	Multiple pathways including storm runoff from dumps, farmlands, landfills, industrial and municipality waste	Plastic additives and adsorbed co-pollutants into water bodies alter the water quality	[64]
All plastics waste	Through inhalation, dietary uptake, drinking water, and using contaminated materials	Humans and animals exposed to microplastics have their health threatened by cytotoxic, genotoxicity, neurotoxicity, oxidative stress, and inflammation	[13,14,64].
All plastic waste	As plastic is obtained from fossil fuels through production, decomposition, degradation, and incineration releases CO_2_	Contribution of greenhouse emissions, carbon footprint, and climate change	[48,52,53]
All plastic waste	Fragments of plastics in seawater contribute to the alkalinity or H adjustment via the release of dissolved organic carbon and other organic acids	Ocean acidification affects marine biodiversity and ecosystems	[55,63]

**Table 4:** Impact of plastic waste on UN SDGs implementation.

Goal	Direct or Indirect Impact of Plastic Waste	Refs
1 No poverty	The detrimental impacts on ecosystem services and subsequent economic repercussions on local communities are major causes of using cheap and affordable plastics.	[11,68]
2 Zero hunger	Microplastics in food packaging, agricultural soils, fruits, vegetables, fish, and shellfish pose potential hazards to human health when consumed.	[11,68]
3 Good health and well-being	Humans, especially fetuses, can absorb microplastics through eating, inhalation, and cutaneous exposure to plastics in food, air, and packaging. This can cause cellular damage and inflammatory and immunological responses	[70]
6 Clean water and sanitation	The detection of traceable microplastics (<5 mm) in potable drinking water and wastewater treatment plants via bottled and sachet water	[71,72,73]
7 Affordable and clean energy	The incineration of (micro)plastic waste in waste-to-energy systems emits greenhouse gases and contributes to air pollution	[75,79]
9 Industry, innovation, and infrastructure	For a circular economy, sustainable bio-based polymers must be innovated.	[75]
10 Reduce inequalities	Plastic waste exports from industrialized to underdeveloped nations are regarded as a form of pollution transmission	[11,76,77]
11 Sustainable cities and communities	Waste plastic hinders urban infrastructure in countries with poor waste management	[78]
12 Responsible consumption and production	Global plastic production and mismanagement	[11,78]
13 Climate action	At each stage of the plastic life cycle, greenhouse gases are emitted from production to waste disposal.	[69]
14 Life below water	Micro-plastic emissions to marine and freshwater ecosystems must be drastically reduced.	[67,73]
15 Life on Land	Micro-plastic waste mismanagement pollutes landfills, urban and rural settings, wildlife habitats, agricultural soils, and terrestrial ecosystems	[11,69]

**Table 5:** Useful fungi and bacteria for microbial degradation.

Source	Enzyme	Microorganism	Plastic as Substrate	Refs
Fungal	Glucosidases	*Aspergillus flavus*	Polycaprolactone (PCL)	[107,112]
		*Penicillium funiculosum*	Polyhydroybutyrate (PHB)	
		*Amycolaptosis* sp.	Polylactic acid (PLA)	
		*Streptomyces* sp.	PHB, PCL	
	Cutinase	*Asperigillus oryzae*	Polybutylene succinate (PBS)	[107]
		*Fusarium* sp.	PCL	
	Catalase, protease	*Aspergillius niger*	PCL	[108]
	Urease	*Trichoderma* sp.	Polyurethane	
	Manganese peroxidase	*Phanerochaete chrysosporium*	Polyethylene	[104]
	Serine hydrolase	*Pestalotiopsis microspora*	Polyurethane	[110]
Bacteria	Lipase	*Rhizopus delenac*	PCL	
		*Rhizopus arrizus*	Polyethylene adipate (PEA), PBS, PCL	[112,113]
		*Firmicutes* sp.	PHB, PCL, and PBS	
		*Protobacteria* sp.		
	Serine hydrolase	*Pseudomonas stutzeri*	Polyhydroxyalkanoate (PHA)	[112]
Fungi		*Aspergillus niger*	Degrades lignin, cellulose, and complex organic materials used for industrial degradation	[114]
Fungi		*Trametes versicolor*	White-rot fungus that degrades lignin and toxic organic pollutants useful for bioremediation	[79]
Fungi		*Phanerochaete chrysosporium*	Effective in degrading phenols, pesticides, and other toxic organic compounds	[108]
Fungi		*Pleurotus ostreatus*	Degrades petroleum-based hydrocarbons, plastics, and agricultural waste	[112]
Fungi		*Mucor mucedo*	Degrades organic compounds including agricultural and food waste	[110]
Fungi		*Bacillus subtilis*	Degrades organic compounds oils and fats applied in waste treatment	[104]
Bacteria		*Alcaligenes eutrophus* and *Rhodococcus rhodochrous*	Degradation of polyclinic aromatic hydrocarbon	[79]
Bacteria		*Geobacilius stearothermophilus*	Degrades organic compounds including petroleum and industrial chemicals at high temperatures	[114]
Bacteria		*Sphingomonas paucimobilis*	Known for the degradation of polycyclic aromatic hydrocarbons (PAHs) used in bioremediation	[115]
Bacteria		*Mycobacterium* species	Breaks down hydrocarbons and PAHs used in the cleanup of oil-contaminated environments	[116]

**Table 6:** Bioremediation of plastics strategies and applications.

Approach	Importance	Microorganism	Application	Refs
Multiomics	Study of metabolomics, metagenomics, proteomics, genomics, and transcriptomics	*Bacillus*, *Pseudomonas*, *Seratia*, *Sphingomonas*, *Halomonas*,	Study the role of genes, proteins, and metabolites involved in bioremediation processes	[105,123]
Biodegradation network	Databases and datasets decrypting degradation information	*Sphingobium*, *Pseudomonas*, *Rhodococcus*, *Sphingonomas*	Present information about recalcitrant chemical compounds in the degradation route	[110,124]
Gene editing tools	Gene knock-out and knock-in experimentation	*Flavobacterium* *Xanthomonas*	Designing the model organisms for bioremediation	[124]
Computational tools	Stoichiometric studies of flux and metabolism of microbe	*Pseudomonas putida*	Mathematical representation of cellular reactions involved in the uptake of substrate and polluting compounds	[105]
Microbial consortium	Fulfill the maximum power principle for enhanced bioremediation	*Brevibacillus* spp. *Vibro* spp.	Designing, optimizing, and constructing synergistic activities of microbial candidates for an increased rate of bioremediation	[125]
Genetic engineering	Combine the function-specific genes of interest for bioremediation study	*Camamonas acidovorans*, *Microbacterium*, *Sphingomonas bisphenolicum*	Optimization and description of genes encoding enzymes and proteins participating in degradation	[115,124]
Enzymatic degradation	Monooxygenases catalyze the desulfurization, dehalogenation, denitrification, ammonification, hydroxylation, biotransformation, and biodegradation of several aromatic and aliphatic chemicals.	*Comamonas acidovorans* strain TB-3 *Nitrosomonas europaea*,	Lignin-degrading enzymes (laccase, manganese-dependent peroxidase) and hydrolases (urease, protease, lipase) for the degradation of various plastic	[100,126]

**Table 7:** Emerging studies on plastic as vectors for many antibiotic-resistant bacteria and genes (ARB and ARGs).

Author	Title	Antibiotic Resistance Genes (ARGs) or Antimicrobial Resistance (AMR) or Antibiotic Resistant Bacteria (ARB)	Remarks on Microplastics (MPs)
[137]	Microplastics act as vectors for antibiotic resistance genes in landfill leachate: The enhanced roles of the long-term aging process	Microbial community evolution and ARGs occurrence of MPs surface during the aging process in landfill leachate	Bacterial communities on MPs showed higher biofilm-forming and pathogenic potential. Aging process could enhance the potential of MPs as vectors for ARGs, which could promote the holistic understanding of MPs behavior and risk in natural environments
[130]	Bacterial Community under the Influence of Microplastics in Indoor Environment and the Health Hazards Associated with Antibiotic Resistance Genes	Redundancy analysis identified specific associations between MP polymers and bacterial taxa, such as polyamide and Actinobacteria	Degradable MPs and nondegradable MPs may result in different health hazards due to their distinct effects on bacterial communities. Also, plastisphere in water and soil environment can affect the bacterial community by enriching ARG
[132]	Microplastics as hubs enriching antibiotic-resistant bacteria and pathogens in municipal activated sludge	Both polyethylene (PE) and polystyrene (PS) microplastics can acclimate biofilms enriched with *sulfonamide* resistance genes	Microplastics can serve as carriers of antibiotic-resistant bacteria (ARB) and pathogens, representing a pressing concern to aquatic biota and human health
[128]	Contribution of microplastic particles to the spread of resistances and pathogenic bacteria in treated wastewaters	A common resistance gene (*sulfonamides*) resulted in being more abundant in the plastisphere than in the planktonic bacterial community	Wastewater plastisphere could promote the spread of pathogenic bacteria and resistance genes in aquatic environment
[135]	Selective enrichment of antibiotic resistance genes and pathogens on polystyrene microplastics in landfill leachate	Mobile genetic elements, bacterial communities, and pathogens on polystyrene in surrounding leachate	MPs could selectively enrich ARGs and pathogens from their surrounding environments,
[116]	Microplastics and Antibiotic Resistance: The Magnitude of the Problem and the Emerging Role of Hospital Wastewater	The interaction of MPs with drug-resistant bacteria and ARGs makes them vectors for the transport and spread of ARGs and harmful microorganisms	MPs represent an ideal substrate for microbial colonization and formation of biofilm, where horizontal gene transfer is facilitated
[129]	Microplastics are a hotspot for antibiotic resistance genes: Progress and perspective	The occurrence and transport of ARGs on MPs in wastewater treatment plants, and aquatic, terrestrial, and air environments were summarized	The enrichment, transport, and transfer of ARGs on MPs, provide a fundamental basis for evaluating their health risk to humans
[134]	Do plastics serve as a possible vector for the spread of antibiotic resistance? First insights from bacteria associated to a polystyrene piece from King George Island (Antarctica)	A polystyrene macro-plastic piece stranded on the shores in King George Island (South Shetlands, Antarctica) was explored with 27 isolated bacterial flora. Kirby–Bauer disk diffusion susceptibility test to 34 antibiotics showed multiple antibiotic resistances against the molecules of cefuroxime and cefazolin, cinoxacin, and ampicillin, amoxicillin + clavulanic acid, carbenicillin and mezlocillin	Results obtained showed MPs can support ARGs even in hostile conditions like the Antarctic area.
[133]	Distinguishing removal and regrowth potential of antibiotic-resistance genes and antibiotic-resistant bacteria on microplastics and in leachate after chlorination or Fenton oxidation	Fate of ARB/ARGs in leachate on MPs treated by chlorination and Fenton oxidation	ARGs/ARB in leachate on MPs exhibited a considerable potential for rapid regrowth after chlorination
[136]	Microplastics in fresh- and wastewater are potential contributors to antibiotic resistance—A minireview	Challenges and consequences of the interaction between MPs and antibiotic-resistant elements in freshwater ecosystem	MPs accumulate and selectively enrich antibiotics, ARB, and ARGs
[131]	Microplastics exacerbate co-occurrence and horizontal transfer of antibiotic resistance genes	Analysis of chicken feces revealed the highest abundance of MPs (14.9 items/g) and ARGs (6.24 × 10^8^ copies/g)	MPs and ARGs in the agricultural environment are dominant in horizontal gene transfer.
[138]	A review of the effect of micro- and nano-plastic pollution on the emergence of antimicrobial resistance	MPs such as plastiglomerates, pyroplastics, and anthropoquinas have become breeding grounds for ARGs and other emerging contaminants (polyaromatic hydrocarbons and pesticides)	Future research perspective on antibiotics and MPs occurrence and environmental impact is highlighted.
[139]	Plastics in the marine environment are reservoirs for antibiotic and metal resistance genes	ARGs and metal resistance genes (MRGs) in microbial communities on the plastics were in the ranges 7.07 × 10^−4^–1.21 × 10^−2^ and 5.51 × 10^−3^–4.82 × 10^−2^ copies per 16S rRNA, respectively	MPs are a reservoir for MRGs and ARGs

## Data Availability

Not applicable.

## Supplementary Materials

The following supporting information can be downloaded at: https://www.mdpi.com/article/10.3390/polym17111521/s1, Table S1. Database search engine and query string results.
