# Supplementary material for: Sustainable Solutions for Plastic Waste Mitigation in Sub-Saharan Africa: Challenges and Future Perspectives Review
Source: Polymers (Basel). 2025 May 29;17(11):1521. doi: 10.3390/polym17111521 (PMC12157109; doi:10.3390/polym17111521)
Supplement: Supplementary file 1 [file polymers-17-01521-s001.zip › polymers-3590358-supplementary.pdf]

# Supplementary Materials

**Table S1.** Database search engine and query string results.

| Database search engine | Enter query string | Query link | Results |
|---|---|---|---|
| Web of science (WOS) | ((((((((((((((((TS=(economic benefits of plastic recycling* )) AND TS=(health benefits of plastic recycling*)) AND TS=(environmental benefits of plastic recycling* )) AND TS=(Africa Plastic recycling*)))) OR TS=(biodegradation of plastic waste*)) OR TS=(bioremediation of plastic waste* )) OR TS=(incineration of plastic waste* )) OR TS=(Plastic recycling in Sub-Saharan Africa)) OR TS=(role of fungi in plastic biodegradation* )) OR ALL=(microbial degradation of plastic waste *)) OR ALL=(role of algae in plastic biodegradation* )) OR ALL=(role of bacteria in plastic biodegradation))) OR KP=(Microplastics degradation | WOS | 2325 |
| Scopus | ALL ((Economic and health benefits) OR (challenges of plastic recycling) OR (environmental benefits of plastic recycling) OR (Africa Plastic recycling) OR (Plastic recycling in Sub-Saharan Africa) AND (biodegradation and bioremediation of plastic waste) OR (incineration of plastic waste) AND (role of fungi and fungi in plastic biodegradation) OR (microbial degradation of plastic waste) AND (role of algae and bacteria in plastic biodegradation) OR (Microplastics degradation)) | SC | 902 |
| Google scholar | Same as Scopus | GS | 487 |
| Total (n) | | | 3714 |

 https://doi.org/10.3390/xxxxx
